# Impact of anxiety levels on the perception of pain in patients undergoing office hysteroscopy

**DOI:** 10.1007/s00404-020-05885-9

**Published:** 2020-11-19

**Authors:** Felice Sorrentino, Annamaria Petito, Stefano Angioni, Francesco D’Antonio, Melania Severo, Maria Cristina Solazzo, Raffaele Tinelli, Luigi Nappi

**Affiliations:** 1 Department of Medical and Surgical Sciences, Institute of Obstetrics and Gynecology, University of Foggia, Viale L. Pinto, 71100 Foggia, Italy; 2 Department of Clinical and Experimental Medicine, University of Foggia, Foggia, Italy; 3 Department of Surgical Science, University of Cagliari, Monserrato, Italy; 4 Department of Obstetrics and Gynecology, “Perrino” Hospital, Brindisi, Italy

**Keywords:** Hysteroscopy, Pain, Anxiety, Stress, VAS

## Abstract

**Objective:**

This study aimed at assessing the impact of anxiety on pain perception during hysteroscopy and to highlight the possible contribution of factors related to pain perception.

**Materials and methods:**

104 women with indication for office hysteroscopy fullfilled anonymous self-report questionnaires during the waiting time, before the procedure. The first self-report questionnaire included general patient information and an overall assessment of the degree of satisfaction with the information received before the procedure. The level of pre-procedural anxiety was measured through the State-Trait Anxiety Inventory STAI-Y1 (state anxiety). The perceived stress was assessed using the Perceived Stress Scale (PSS). The intensity of pain during the procedure and 20 min later was assessed with VAS score.

**Results:**

The average waiting time was of 192.33 ± 91 min. 59 patients (56.7%) performed the examination without analgesia while 45 women (43.3%) required analgesia. 28 women (27%) experienced mild pain, 34 (33%) moderate pain and 42 (40%) severe pain. The patients who performed the procedure without analgosedation had an average STAI-Y1 score of 44.81 ± 1.20, compared to women who required analgosedation (average score of 49.40 ± 1.64). The perceived level of stress was also associated with the use of analgosedation. Patients who did not request any anesthetic intervention obtained a PSS average score of 16.66 ± 0.75, compared to the subgroup with anesthesia (score of 19.76 ± 0.90).

**Conclusions:**

Anxiety represents a key element for the success of ambulatory hysteroscopy. The management of anxiety can reduce the request for analgesia with a consequent optimization of time, costs and safety.

## Introduction

The development of hysteroscopy has provided a minimally invasive approach to common gynecologic problems. Increased clinician training, smaller diameter hysteroscopes and increased emphasis on office-based procedures have led to a widespread use of this important technology. Hysteroscopy is an endoscopic procedure that allows direct visualization of the uterine cavity and has become the gold- standard in the diagnosis and treatment of many gynecological pathological conditions like infertility, AUB, uterine malformations, cervical and vaginal disease. The real revolution in hysteroscopy took place with the introduction of the outpatient procedure that allows the reduction of the risks associated with anesthesia, reduction of costs and duration of the procedure with better patient compliance. The use of a more advanced instrumentation has also allowed to concentrate diagnostic and operative times in a single clinical moment, defined as "See and Treat" [1–4].

Currently, the main limiting factor for a widespread use of the technique in an outpatient setting is the pain and discomfort felt by patients which can cause up to 84% of failures [5, 6].

The variables associated with the perception of pain during hysteroscopy may be related to the technique used or the characteristics of the patient.

Nulliparity, menopause, chronic pelvic pain, a previous cesarean section, the duration of the procedure and the age of the patient were all associated with higher pain perception [7], while the use of saline solution instead of CO_2_, a smaller instrument, the vaginoscopic approach, the operator’s skills and the non-touch technique, were all associated with less pain perception and/or less administration of anesthesia [2, 8–11].

Anxiety plays a peculiar role in the modulation of pain, through central and peripheral neurobiological mechanisms and represents an important patient-related variable, still underestimated, that can negatively influence the tolerability of the procedure.

This aspect would explain why, despite the progressive simplification of the technique and the non-need for anesthesia, the gap between the theoretical invasiveness of the procedure and the patients' expectations remains unresolved [12].

Pain is not just a problem itself, because it is related to the success of hysteroscopy, both diagnostic and operative [13]. Despite its importance, the role of anxiety and its management in office hysteroscopy remains still not well investigated.

Our study aims to assess the impact of anxiety on pain perception during hysteroscopy in an outpatient setting and to highlight the possible contribution of factors related to pain perception.

## Material and methods

### Participants

Between October 2018 and March 2019 104 women with indication for office hysteroscopy were admitted to Department of Obstetrics and Gynecology of University of Foggia. All patients were informed about the procedure and the possibility to ask for analgosedation during surgery.

### Measures

After obtaining informed consent the patients were asked to fullfill anonymous self-report questionnaires during the waiting time, before the start of the procedure (the time between the completion of the questionnaires and the beginning of the procedure). The first self-report questionnaire included general patient information: marital status, level of education, age, gynecological anamnestic data. In addition, an overall assessment of the degree of satisfaction with the information received before the procedure, choosing between “very good, good, enough, poor, very poor”.

The level of pre-procedural anxiety was measured through the State-Trait Anxiety Inventory (STAI). STAI is considered the first instrument that allows to evaluate trait anxiety and state anxiety separately. State anxiety is defined as a transient emotional state or as a condition characterized by subjective feelings perceived on a conscious level of tension and apprehension and by increased activity of the autonomic nervous system. It may vary and fluctuate over time [14].

Trait anxiety, instead, refers to relatively stable individual differences, in the disposition towards anxiety, for example differences between people in the tendency to respond with elevation of the intensity of state anxiety to situations perceived as threatening. With trait anxiety we mean a general attitude in perceiving anxiety, while with state anxiety we mean a situational anxiety, circumstantial over time.

The State-Trait Anxiety Inventory (Form Y) is a self-assessment test consisting of 40 items, 20 for state anxiety (Y1) and 20 for trait anxiety (Y2). The subject must respond in terms of intensity based on a 4-point Likert scale (1–2–3–4) [15].

The STAI-Y1 items refer to current feelings perceived "at this time", the subject expresses a frequency rating through a score from 1 a 4, where: 1 = not at all; 2 = a little; 3 = enough and 4 = very much. The Y2 form investigates how the subject usually feels, the subject expresses an evaluation on a Likert scale with scores from 1 to 4, where: 1 = almost never; 2 = sometimes; 3 = often and 4 = almost always.

The scoring range for each test is 20–80 with a cut-point of 39–40. In the form Y1 to the items 1, 2, 5, 8, 10, 11, 15, 16, 19, 20 the reverse score is attributed, so if the degree of agreement is 1, the value 4 is attributed, if the degree of agreement is 2, it is attributed the value 3 and if the degree of agreement is 3, it remains unchanged; the value of 1 corresponds to the degree of agreement 4. For the rest of the items, is provided a direct attribution of a score from 1 to 4 based on the point on the Likert scale marked. In the Y2 form the reverse items are 1, 3, 6, 7, 10, 13, 14, 16, 19. The perceived stress was assessed using the Perceived Stress Scale (PSS) [16].

The items were built to assess how people find their lives unpredictable, uncontrollable or overloaded.

The scale also contains a series of direct questions on current levels of perceived stress. The PSS was designed for samples of general population with a school level of at least middle school. Both the items and the alternatives are easy to understand. Furthermore, the questions are of a general nature and, therefore, are free from specific contents of some subpopulation. PSS questions are about feelings and thoughts related to the last month.

For each question, subjects are asked to indicate how often they felt in a certain way, through a score from 0 to 4, where 0 = Never; 1 = Almost never; 2 = Sometimes; 3 = Quite often and 4 = Very often.

PSS scores are obtained by reversing the scores (for example, 0 = 4, 1 = 3, 2 = 2, 3 = 1 and 4 = 0) given to the four positively formulated items (items 4, 5, 7 and 8) and then adding up all the items on the scale. It has been shown that PSS correlates with the following: stress measures, measures related to health and health services, measures on healthy behaviors, smoking-related habits, help-seeking behavior [17]. The time between the completion of the questionnaires and the beginning of the procedure was assessed as waiting time.

### Procedures

All office hysteroscopic procedures (see Table 2) were performed by one experienced hysteroscopist by means of a 4-mm continuous-flow office hysteroscope (Bettocchi Office Hysteroscope “size 4” Karl Storz, Tuttlingen, Germany) with a 2.9-mm rod lens optic. The vaginoscopic approach was used; no analgesia or anesthesia was administered to the patient at the beginning of procedure. In case of discomfort or pain during surgery on patient request intravenous (IV) drugs (propofol 1 mg/kg and/or fentanyl 0.05 mg) were administered through intravenous catheter.

For the distension of uterine cavity we used saline solution and the intrauterine pressure was automatically controlled by an irrigation–suction electronic device (Endomat; Karl Storz) set at 45 mm Hg. When needed, operative procedures were performed using traditional and second-generation techniques as previously described [18–20]. At the end of the procedure, the patients remained under observation for 30 min.

It was also requested to assess the intensity of the pain perceived during the procedure and 20 min later, using a Visual Analogue Scale (VAS), which consisted of a printed 100 mm horizontal line anchored by the descriptors “no pain” (minimum, on the left end of the scale) and “worst pain imaginable” (maximum, on the right end). VAS score was not evaluated 20 min later in the patients who requested analgosedation in order to avoid unreliable data related to the use of intravenous drugs and their effects on patients.

All data were analyzed with IBM SPSS Statistics 25.0. Quantitative variables are expressed as mean and standard deviation (SD).

Subgroups of patients were compared using unpaired samples *t* test. *P* < 0.05 was considered statistically significant.

## Results

**Fig. 1:**
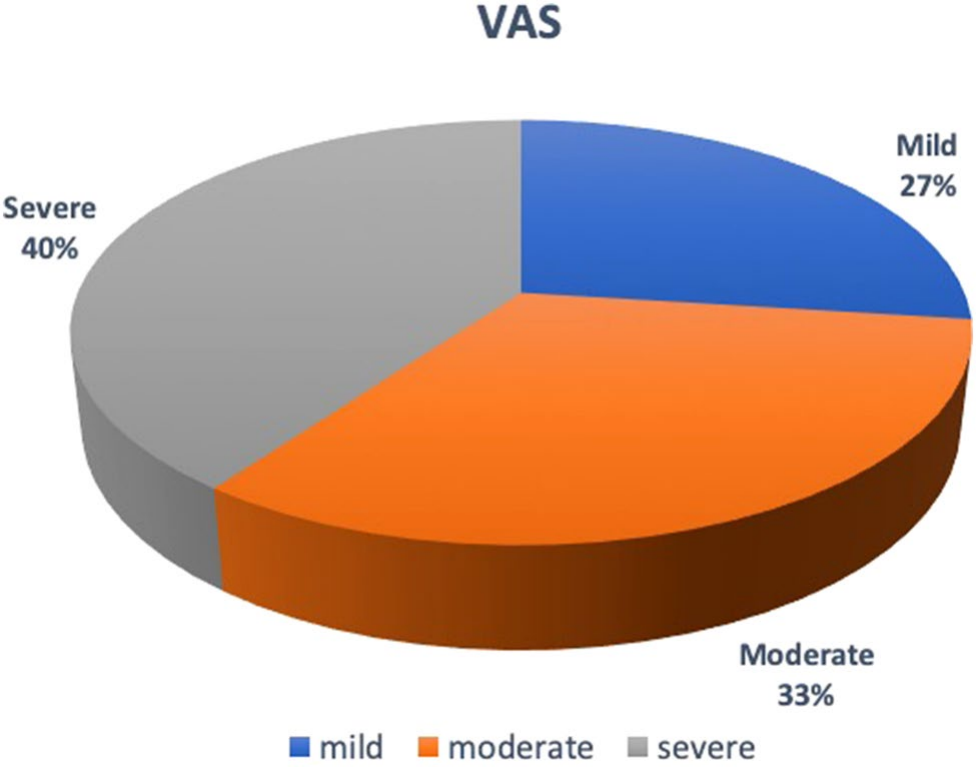
VAS score

104 consecutive patients were enrolled in this study. The characteristics of this sample are shown in Table 1. The average waiting time was of 192.33 ± 91 SD minutes. During the procedure 59 patients (56.7%) performed the examination without analgesia while 45 women (43.3%) required analgesia. The data of the two groups were compared. As reported in Fig. 1, 28 women (27%) experienced mild pain (VAS = 0–30), 34 women (33%) felt moderate pain (VAS = 40–70) and 42 (40%) women severe pain (VAS = 70–100).

**Table 1:** Characteristics of total sample (104)

Variables	
**Age (M ± SD)**	49.30 ± 12.26
**Educational level ** ***n*** **(%)**	
Elementary Middle High Graduation	11 (10.6%) 20 (19.2%) 48 (46.1%) 25 (24.1%)
**Marital status ** ***n*** **(%)**	
Single Married Widower Divorced	21 (20.2%) 65 (62.5%) 7 (6.7%) 11 (10.6%)
**First hysteroscopy ** ***n*** **(%)**	72 (69.2%)
**Level of information about the procedure ** ***n*** **(%)**	
Very good Good Enough Poor Very poor	8 (7.7%) 43 (41.3%) 28 (27%) 17 (16.3%) 8 (7.7%)
**Menopause ** ***n*** **(%)**	
Yes No	47 (45.2%) 57 (54.8%)
Nulliparous *n* (%)	28 (26.9%)
Multiparous *n* (%)	76 (73.1%)
Vaginal birth *n* (%)	50 (48%)
1 previous CS *n* (%)	18 (17.3%)
Miscarriage *n* (%)	30 (28.8%)

In relation to the level of anxiety and perceived stress, the STAI-Y1 average score was 46.75 ± 10.48 SD, the STAI-Y2 average score was 39.86 ± 9.43 SD and the PSS score was 18 ± 6.06 SD.

In the non-analgesia group the average waiting time was 174.7 ± 91.94 SD. These women obtained an average STAI-Y1 score of 44.80 ± 9.35 SD; STAI-Y2 of 38.67 ± 8.58 SD, PSS of 16.65 ± 5.78 SD and reported a mean VAS score during the procedure of 43.05 ± 27.86 SD and 14.20 ± 20.92, 20 min after the procedure.

Six women (10.2%) reported that the information received about the procedure was very good, 27 patients (45.8%) rated it as good, for 15 patients (25.4%) it was enough, for nine women (15.2%) it was poor and for two women (3.4%) it was very poor.

In the analgesia group the average waiting time was 216 ± 84.5 SD minutes.

**Fig. 2:**
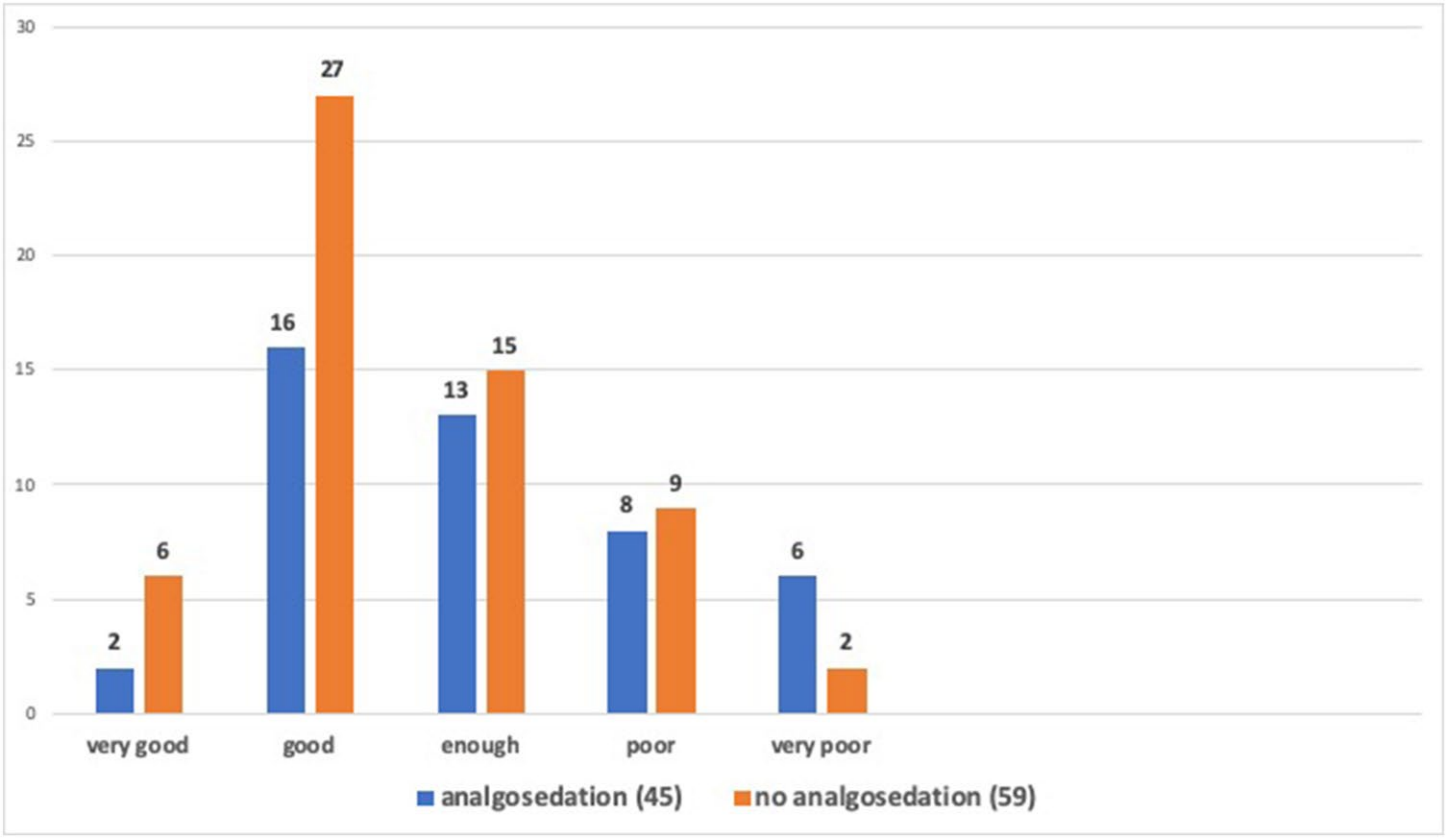
Quality of information received by patients

The average scores of the state of anxiety and perceived stress were, respectively, STAI-Y1 of 49.40 ± 11.32 SD, STAY-Y2 of 41.52 ± 10.28 SD and PSS of 19.75 ± 5.97 SD. The request for analgesia was associated with a high level of pain intensity with a mean VAS score of 66.54 ± 26. All average scores were higher than the previous subgroup. These patients reported the quality of the information received as very good in 4.4% of the cases (2 of 45 women), 16 (35.5%) judged them to be good, for 13 (28.9%) patients the information received was enough, for 8 (17.8%) women the information was poor and for 6 (13.4%) very poor (Fig. 2). Table 2 compares the average scores of the different variables in the two subgroups of the sample.

**Table 2:** The average scores of the different variables in the two subgroups

Variables	Analgosedation, *N* 45 (43.3%)	No analgosedation, *N* 59 (56.7%)
VAS (*M* ± SD)	66.54* ± 26	43.05 ± 27.86
VAS after 20 min (*M* ± SD)	**	14.20 ± 20.92
STAI Y1 (*M* ± SD)	49.40 ± 11.32	44.80 ± 9.35
STAI Y2 (*M* ± SD)	41.52 ± 10.28	38.67 ± 8.58
PSS (*M* ± SD)	19.75 ± 5.97	16.65 ± 5.78
Waiting time (*M* ± SD)	216 ± 84.5	174.7 ± 91.94
Hysteroscopic procedures *n* (%)	25 polypectomies (55.6%) 10 diagnostics (22.2%) 8 endometrial biopsies (17.8%) 1 metroplasty (2.2%) 1 myomectomy (2.2%)	24 polypectomies (40.7%) 16 diagnostics (27.1%) 15 endometrial biopsies (25.4%) 1 metroplasty (1.7%) 2 myomectomies (3.4%) 1 intrauterine device (IUD) removal (1.7%)

*Value calculated on the number of patients before they required analgosedation

**Not evaluated in these group to avoid unreliable data related to the use of intravenous drugs

The correlation between the scores obtained with STAI-Y1 and VAS was analyzed with the Pearson correlation coefficient for both subgroups in the sample. Furthermore, the correlation of STAY-Y1 values and the degree of overall patient satisfaction was also analyzed.

**Fig. 3:**
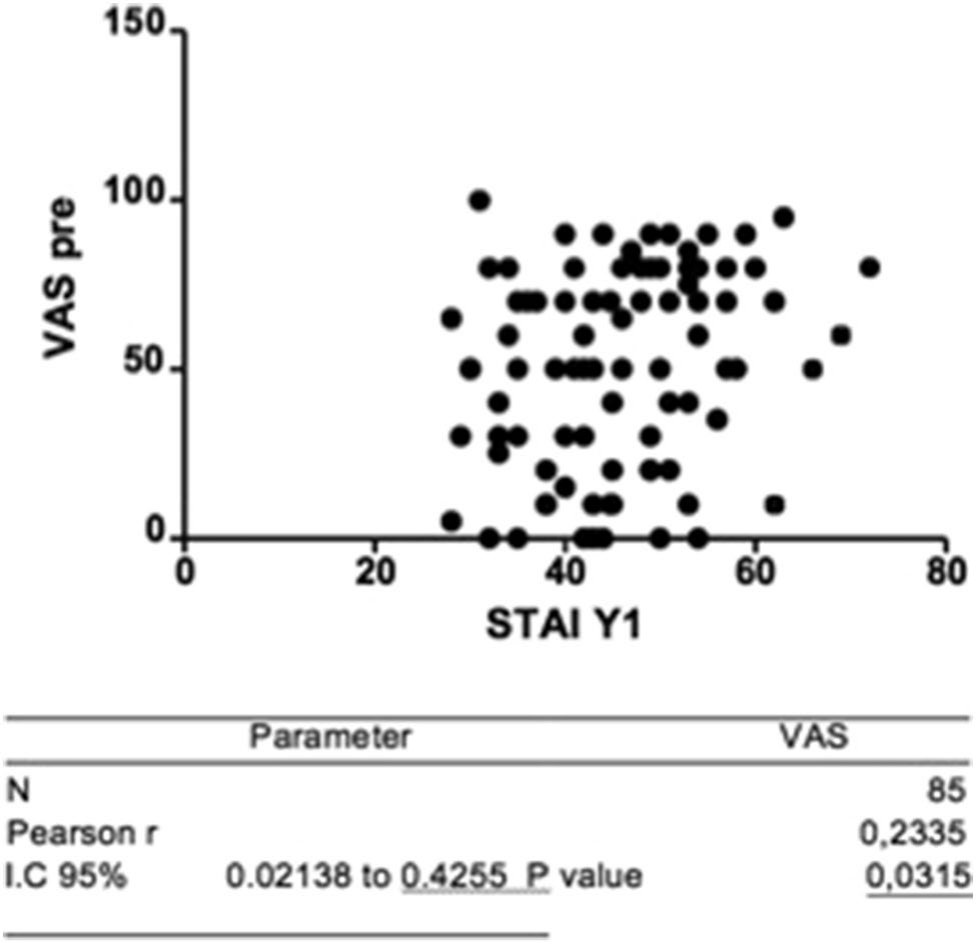
Pearson correlation coefficient between STAY-Y1 e VAS

**Fig. 4:**
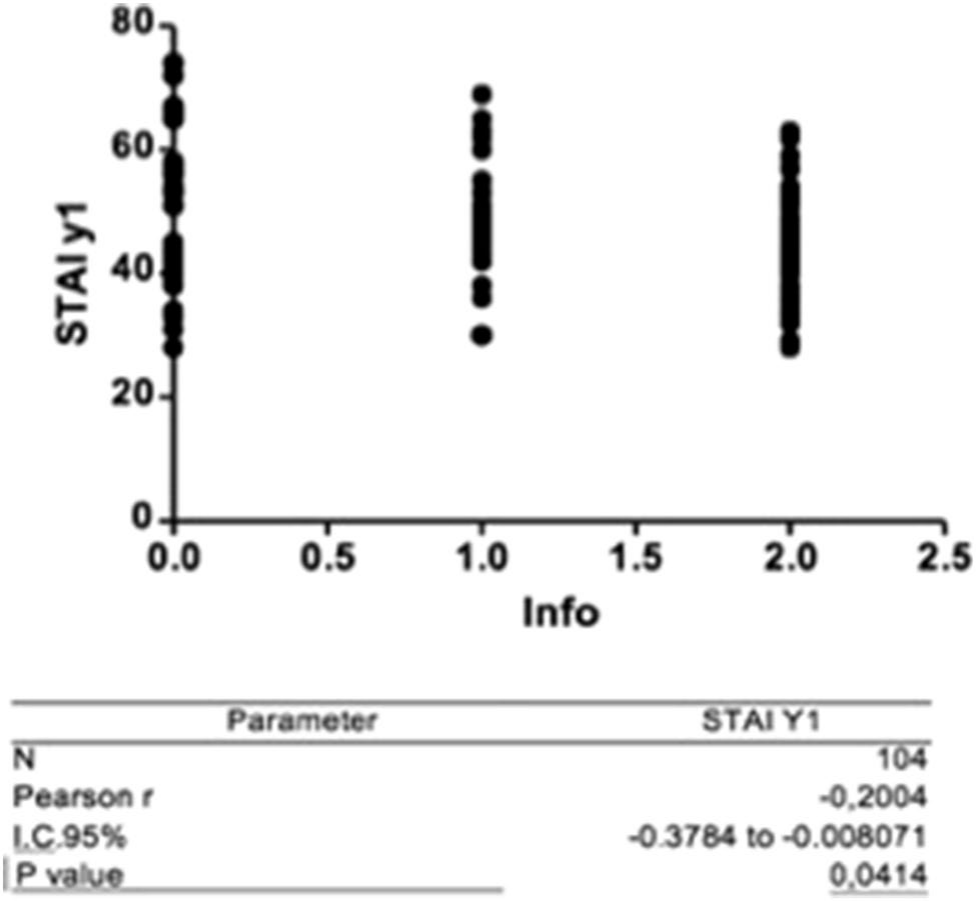
Pearson correlation coefficient between STAI-Y and information received

The analysis showed a positive correlation between STAI-Y1 and VAS scale (*r* = 0.2; *p* < 0.05), indicating that patients with greater state anxiety perceive greater pain intensity during hysteroscopy. A negative correlation also emerged between the STAI-Y1 and the judgment on the information received before the examination (*r* = − 0.2; *p* < 0.05), highlighting a greater level of state anxiety in patients who negatively evaluated the information received. The results of these correlations are shown in Figs. 3 and 4.

*T* test was used to evaluate the difference between the average scores obtained in the two subgroups of the sample. The first analysis shows that the patients who performed the procedure without analgosedation had an average STAI-Y1 score of 44.81 ± 1.20, compared to women who required analgosedation who obtained an average score of 49.40 ± 1.64.

**Fig. 5:**
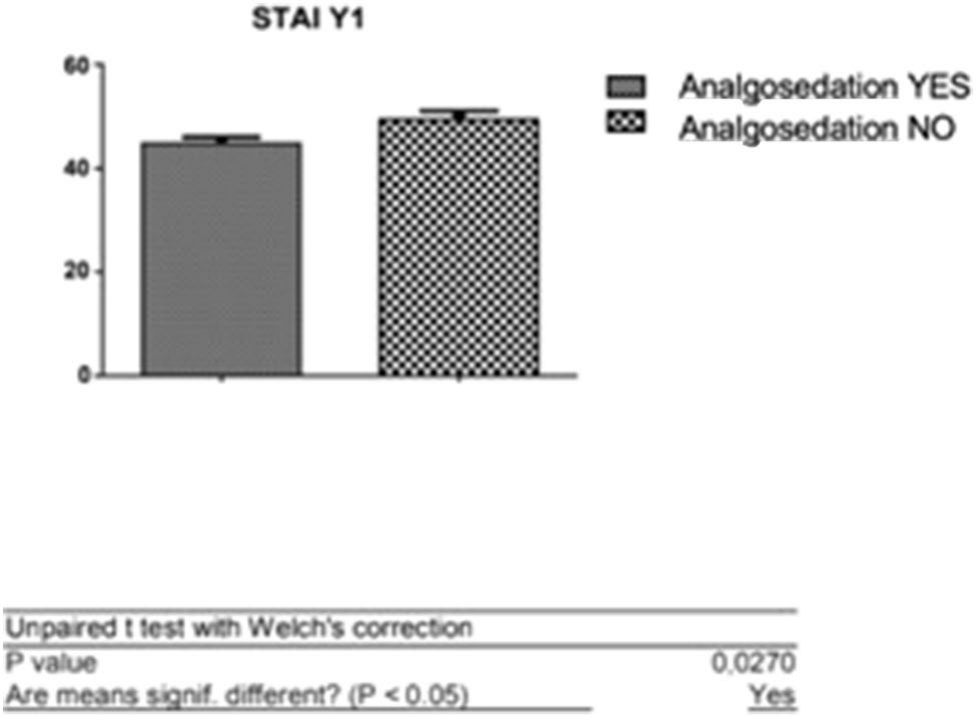
*T* test: analysis of the difference between averages of scores obtained at STAI-Y1 (patients with and without analgosedation)

This shows how patients with higher STAI-Y1 scores are more likely to require analgesia (Fig. 5).

The perceived level of stress was also associated with the use of anesthesia; in fact there was a significant difference between the averages PSS scores in the two subgroups.

**Fig. 6:**
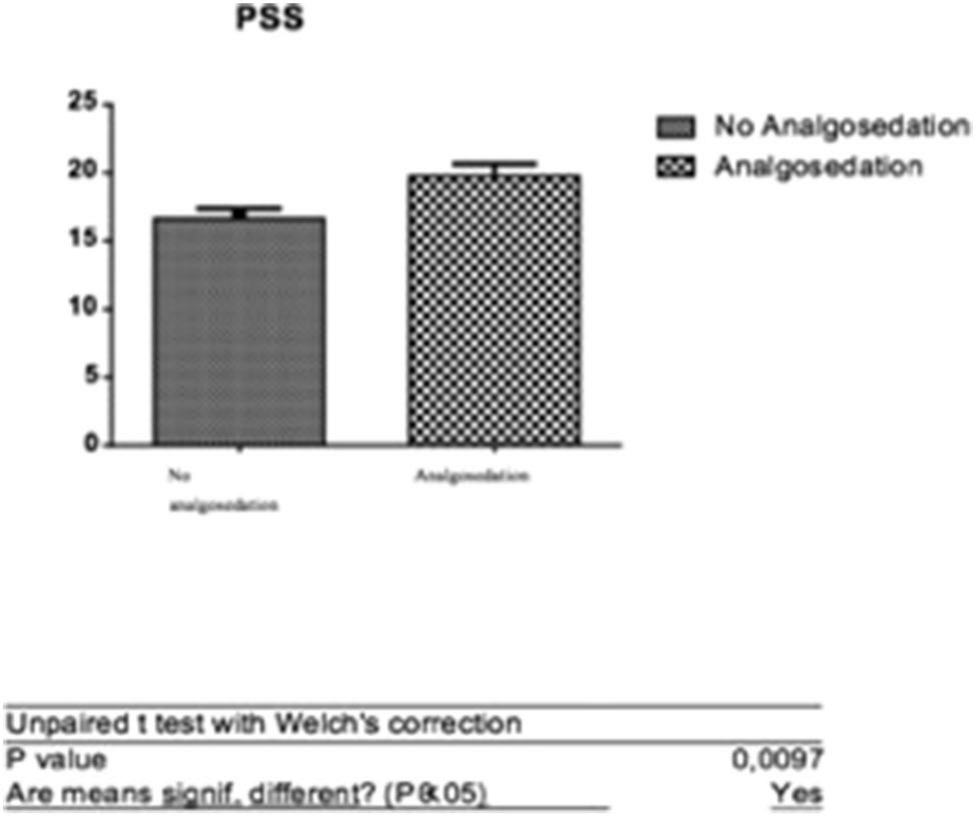
*T* test: analysis of the difference between averages of PSS scores (patients with and without analgosedation)

In particular, patients who did not request an anesthetic intervention obtained a PSS average score of 16.66 ± 0.75, compared to the subgroup with anesthesia with the score of 19.76 ± 0.90 (Fig. 6).

**Fig. 7:**
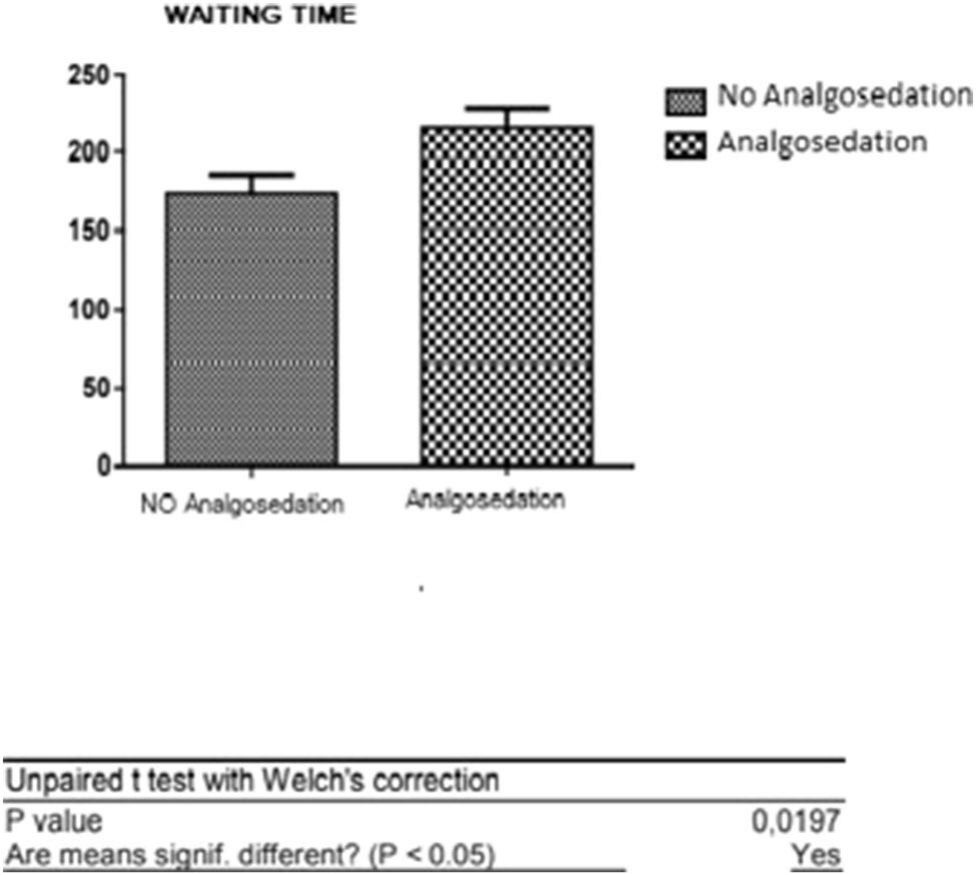
*T* test: comparative analysis between waiting time for patients with and without analgosedation

As shown in Fig. 7, in the two subgroups there was a difference in the average waiting time: patients who did not receive anesthesia waited an average of 174.7 ± 91.94 min before starting the hysteroscopic examination, compared to the second subgroup that had an average waiting time of 216 ± 84.5 min.

## Discussion

The descriptive analysis of the sample revealed that 73% of women undergoing hysteroscopy felt moderate to severe pain (VAS = 40–100). So pain can be considered as the main factor limiting the spread of hysteroscopy in an outpatient setting.

However, we have to underline that acceptability of the endoscopic examination should not only consider the factors related to technical characteristics, but also those patient-related variables. Our study showed that anxiety is significantly associated not only with higher levels of perceived pain, but also with a greater probability of requiring analgosedation during the procedure.

In particular, the STAI-Y1 mean score of 46.75 ± 10.48 SD was higher in the total sample than the one recorded in general population [21]. High levels of anxiety in patients awaiting hysteroscopy were also recorded in other studies. In a large Italian study published in 2007, 65% of the 533 women interviewed by a doctor, before hysteroscopy, reported preoperative anxiety (an unpleasant state of discomfort or tension) [7]. However, in this study anxiety was assessed by asking the patient a simple question, effectively invalidating the measurement. Other authors have attempted to measure pre-hysteroscopic anxiety with structured and validated methods. One study measured the anxiety levels of 30 women before outpatient hysteroscopy, reporting an average anxiety level of 46.07 (± 11.39 SD), using the state anxiety index (STAI) [22].

Gupta et al. reported data related to state anxiety of 240 women attending a see-and-treat outpatient hysteroscopy clinic and compared them with women in other clinical situation [23].

The levels of anxiety before hysteroscopy were significantly higher than those measured in 73 women attending a general gynecological clinic, while similar levels were found in patients present in the clinic for chronic pelvic pain [23].

It is interesting to note that patients seem to have higher levels of anxiety than women undergoing other medical procedures, considered to be more invasive [24] and that the anxiety experienced before hysteroscopy is comparable to that of women undergoing gynecological major surgery under general anesthesia [25, 26].

This clearly suggests a significant gap between the clinical view of what is minimally invasive and the patient's expectation.

With regard to perceived anxiety, our study showed that patients of both subgroups experience elevated state anxiety, understood as a feeling of tension influenced by the current situation and generally related to the uncertainty of future events. Statistical analyses show that high levels of STAI-Y1 are associated with a greater probability of requiring analgosedation (*p* < 0.05). The result could be explained considering the statistically significant correlation between STAY-Y1 levels and VAS scores (*r* = 0.2; *p* < 0.05) found in the sample. Higher levels of state anxiety are related to greater pain intensity and this may cause the request of analgosedation.

Several studies have highlighted how pain perception can be modulated by non-organic factors such as anxiety. In particular, two different works affirm that the tolerability threshold of sternal and myofascial pain can be influenced by the emotional state of patients [23, 27]. The neuro-biological mechanisms that can explain our results are based on the assumption of a biological interconnection between the physiological effects of the anxiety and the perception of pain, both centrally and peripherally. Among the range of negative emotions capable of modulating pain, anxiety certainly plays an important role.

Our study also showed that even higher stress levels are related to a greater probability of requiring analgosedation. Patients who did not request an anesthetic intervention obtained an average score of 16.66 ± 0.75 at the PSS, compared to the second sub-group that achieved a score of 19.76 ± 0.90.

The result is in agreement with several clinical studies showing the role of stress in pain modulation. In fact, stress would seem to reduce the pain tolerance threshold in children with abdominal pain, in subjects with fibromyalgia and in patients suffering from gastro-esophageal reflux. From these data it emerges how stress management techniques could represent a new key to pain management [28].

It seems clear that anxiety is a key factor on pain modulation, although the hypotheses that attempt to explain the rational may be complex.

The knowledge of this information represents a crucial starting point for pain management in clinical practice, especially during those medical procedures that do not necessarily require analgesia or anesthesia. In this perspective, our study has shown that waiting time and the quality of information offered to patients are also correlated to a greater probability of requiring anesthesia and to higher levels of anxiety.

In the two subgroups of our sample there was a significant statistical difference between the waiting time averages: 174.7 ± 91.94 min for patients who performed the examination without analgosedation and 216 ± 84.5 SD minutes for patients who required analgosedation. So we could state that the reduction of waiting time represents a valid strategy not only for the reduction of the discomfort experienced by patients and for an improvement in compliance, but also for a reduction of the risks associated with analgosedation, recovery times and finally optimization of costs. The goal is to increase tolerance to pain by managing anxiety through psychological techniques or pharmacological agents. Some authors describe the routine administration of anxiolytics; however, considering the generally short duration of the procedure and the possible side effects of the oral medication, it would be preferable to use non-pharmacological instruments and it would also be more in line with the philosophy of the minimally invasive hysteroscopy office [12].

Communication with patient and education can play an important role in the management of pre-operative anxiety. In our sample a negative correlation emerged between the STAI-Y1 scores and the patients' overall judgment about the information received from the healthcare staff (*r* = − 0.2; *p* < 0.05). Women who considered the degree of information poor or very poor before undergoing the procedure had higher levels of anxiety. It has been shown that preoperative anxiety is effectively reduced by the ability of doctors to answer patients' questions, which also increases patient satisfaction [29].

The difficulty of effective preoperative communication could be overcome with multidimensional approaches that seem to improve patient understanding and satisfaction. Considering that women are conscious during office hysteroscopy, communication should also continue during the procedure, informing patients of the progress of the procedure [30].

We also evaluated clinical variables that could be considered predictors of pain during office hysteroscopy (parity and menopausal status). The rationale of this hypothesis is based on the relationship between the size of the cervix and the diameter of the hysteroscope (the cervical canal and the smaller internal orifice in the nulliparous patients would cause a more difficult passage of the hysteroscope and therefore more pain). However, in our study none of these variables were related to request of analgosedation or to higher VAS scores as previously demonstrated by other authors [31].

**Fig. 8:**
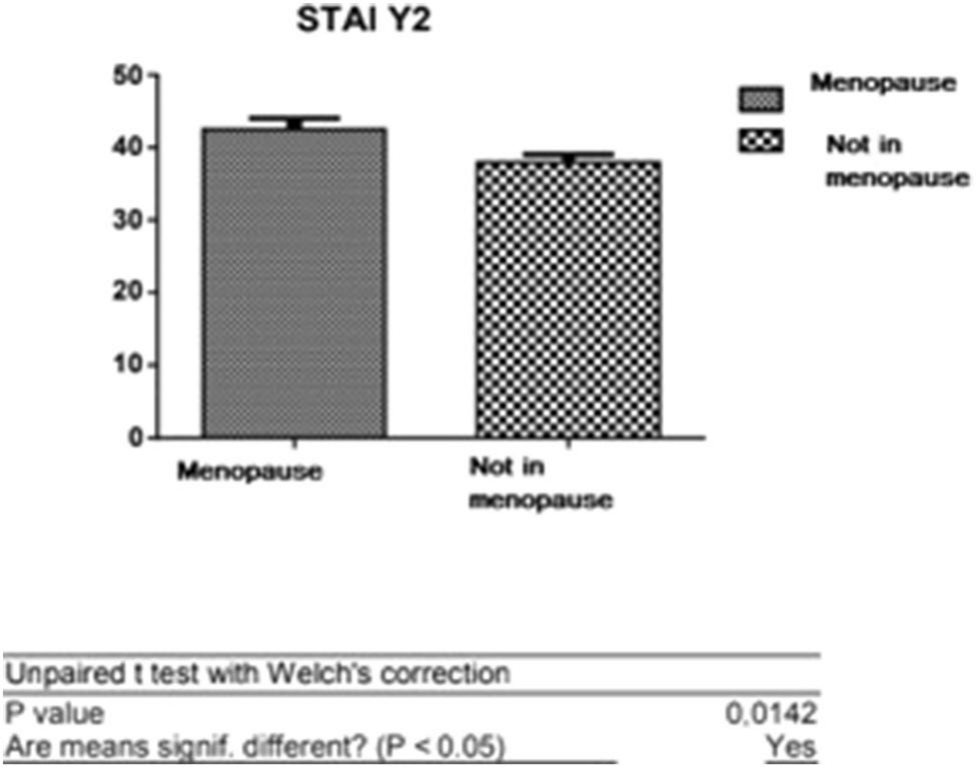
*T* test: analysis between the STAI Y-2 average scores between menopausal and non-menopausal patients

The study sample showed a significantly higher average of STAY-Y2 scores in menopausal women than in non-menopausal patients Fig. 8. The data are not related to the objective of the study but can be explained considering the hormonal estrogenic deficit characteristic of post-menopause.

## Conclusions

Our study shows how anxiety before an outpatient procedure represents a key factor in the perception of pain and also the gap between what is clinically considered non-invasive and how the patients actually experience the procedure. Women undergoing ambulatory hysteroscopy experience significant levels of preoperative anxiety, comparable to those experienced before more invasive procedures under general anesthesia.

Anxiety, by influencing the perception of pain, represents a key element for the success of the procedure, the general satisfaction of the patient and the use of analgosedation.

Our results have important clinical implications because the management of anxiety can reduce the request for analgesia with a consequent optimization of time, costs and safety.

Among the limitations of our study we can include the relatively small number of the sample and the use of tools for evaluating anxiety and self-report stress.

Future studies might use semi-structural interviews and the direct measurement of biological parameters related to anxiety before and after the procedure.

Also it would be very important to investigate the role of non-pharmacological interventions in reducing the experience of anxiety in hysteroscopy (patient education, communication, psychological support and relaxation techniques through traditional or multimedia approaches).
